# Determinants of carbon emission: A multiple scale decomposition of Gansu Province

**DOI:** 10.1371/journal.pone.0309467

**Published:** 2024-09-06

**Authors:** Yanan Wang, Jingxin Xie, Liwen Fu, Baljeet Singh

**Affiliations:** 1 School of Economics and Finance, Hohai University, Changzhou, China; 2 The University of the South Pacific, Suva, Fiji; Jinan University, CHINA

## Abstract

China, being the largest contributor to total carbon emissions, still has a long way to go in energy conservation and emission reduction. Employing the structural decomposition analysis (SDA) method and using input-output table data, this study examines the evolution of carbon emissions resulting from energy consumption in Gansu Province in China over the period 2007 to 2017. By exploring carbon emission driving factors and identifying key final demand and sectors for carbon emissions, Gansu province can formulate more effective emission reduction policies that can balance economic development and carbon emission control. The key findings are as follows: 1) Regarding the driving factors, both the energy intensity effect and the demand sector structure effect emerge as the main contributors to emission reduction. Conversely, the total demand effect and the input-output structure effect predominantly led to emission increase. 2) In terms of each final demand, urban residents’ consumption, rural residents’ consumption and outflow represent the primary categories contributing to increased emissions. 3) The sectors experiencing the most significant decline in carbon emissions and carbon intensity are Electricity, Heat Production and Supply Industry, while Metal Smelting and Rolling Processing Industry as well as Construction Industry are the primary contributors to increasing emissions. Consequently, to achieve the carbon neutrality goal, Gansu governments should consider all these factors and propose mitigation policies in light of the local realities.

## 1. Introduction

The critical challenge of climate change poses a profound threat to human survival, economic growth and social development [1]. Since the enactment of the United Nations Framework Convention on Climate Change in 1992, nations worldwide have implemented a series of measures to conserve energy and reduce emissions [2]. Achieving China’s carbon neutrality target holds great significance for promoting the harmonious development of all humanity and the ecological environment. Consequently, the Chinese government has actively advocated for the long-term planning and implementation of carbon emission reduction policies [3]. However, in the current stage of economic transformation, China’s total carbon emissions are still closely and positively correlated to economic development [4,5]. Most provinces have not yet achieved the decoupling of economic development from carbon emission pressure [6]. Given this situation, the top priority to achieve China’s low-carbon development is the optimisation of industrial structure and advancement of sustainable economic practices [7]. More than 90% of China’s total carbon emissions are caused by energy usage, and most of this energy usage emission is caused by energy-producing provinces. Specifically, the total carbon emissions of Inner Mongolia, Xinjiang, Shanxi, Shaanxi and Gansu alone account for about 30% of the national carbon emissions. Therefore, specific focus should be placed on analysing major energy-consuming provinces when exploring the factors influencing carbon emission reduction. Gansu Province, rich in metal minerals and energy minerals, has become a crucial base for metal raw materials and basic energy supply in China. Subsequently, Gansu Province has gradually developed an industrial structure dominated by heavy industries depending on energy and mineral resources [8]. With the expansion of key industries, the environmental situation in Gansu Province has worsened, leading to serious air pollution [9].

Given the above background, this study focuses on Gansu Province, a representative economically undeveloped province in the western region and a province characterised by its significant energy and resource intensity. Utilising input-output table data, this paper uses the structural decomposition analysis method (SDA) to analyse the total carbon emissions caused by energy use. The objective is to probe into the driving forces influencing changes in carbon emissions and to examine the interplay among various industrial sectors. The implied carbon emissions calculated by using the input-output table considered the carbon emissions resulting from the utilisation of products from other departments during production. This approach provides a more comprehensive perspective, better reflecting each sector’s accountability in terms of energy conservation and emission reductions.

The innovations and contributions of this study are as follows: 1) The study uses unique data set from Gansu Province. Gansu Province, a representative economically underdeveloped province and energy-intensive region in western China, was chosen as the primary case study to provide insight on sustainable development of economy and energy in similar regions in China and Asia. The task of achieving carbon emission mitigation through industrial restructuring, transforming and upgrading is particularly arduous for underdeveloped and energy-intensive regions in developing countries. Thus, analysing the driving factors of carbon emission in least-developed provinces can provide great insight to developing countries to realise its carbon peak and neutrality target. The study is based on Gansu Province mainly due to availability relevant data required for the study. 2) This paper adopts the SDA method and selects decomposition factors at multiple scales. It delves into the fundamental drivers of carbon emission changes in Gansu province across six dimensions: energy structure, energy intensity, input-output structure, demand sector structure, demand type structure and total demand. 3) This study combines macro and micro perspectives, examining both at the province level and specific sector level within Gansu province. It explores the driving factors of carbon emission changes from diverse viewpoints. The findings provide critical insights to guide policy makers in designing fine-grained carbon mitigation policies at different levels in China and in other least developed energy-based economies or regions facing similar challenges. In addition, lessons learned from studying least-developed provinces could inform international efforts to address climate change and reduce carbon emissions on a larger scale [10].

The remainder of the study is ordered as follows. Firstly, section 2 summarises the extant literature on drivers of CO_2_ emissions. Secondly, section 3 provides step-by-step details of the research methodology. Thirdly, section 4 outlines details of data sources and data processing. Finally, section 5 lists the findings and discusses the results. The final section of the paper provides the policy recommendation.

## 2. Literature review

Reducing carbon emissions in China is a formidable phenomenon. In the 75th session of the United Nations General Assembly in 2020, China proposed carbon peak and neutrality targets. Most scholars believed that China can fulfill its carbon emission reduction commitments and this conclusion was reached by modeling and projecting China’s total carbon emissions, carbon emission intensity and the trajectory of carbon emissions [10–12]. The Government is committed to improve the effectiveness of environmental regulation implementation, fully utilise the various environmental regulation tools to achieve carbon neutrality [13] and correctly address the relationship between fiscal decentralisation and environmental governance performance [14].

However, key drivers behind carbon emissions have consistently been debated in academic discourse. To identify factors influencing changes in carbon emissions, scholars in the academic field have employed methodologies such as econometrics and factor decomposition analysis among other methods. Traditional econometric methods treat research units as independent and homogeneous entities [15]. Nevertheless, the ongoing development of spatial econometrics provides an effective approach for examining the phenomenon characterised by geospatial heterogeneity. Wang et al. [16] constructed a spatial difference-in-difference (SDID) model to analyse the spatial spillover effect and driving mechanism of NEDC policy on urban carbon emissions. China’s rapid economic growth is the main factor causing the increase of total carbon emissions [17,18]. In addition, urbanisation, energy intensity, transportation and other factors have been found to have an impact on China’s carbon emissions [19–21]. At the same time, scholars found that carbon emissions can be reduced through technological progress, reduction of energy intensity, improvement of emission efficiency and enhancement of carbon emission time heterogeneity [22–24]. However, the spatial econometric model requires high data for the analysis of factors affecting carbon emissions, and ignores the impact of the time dimension, so it is unable to effectively decipher the cause and effect behind the geographical phenomena. In contrast, factor decomposition analysis is more suitable for geographical phenomena with temporal characteristics.

The mainstream factor decomposition analysis includes Index Decomposition Analysis (IDA) and Structural Decomposition Analysis (SDA). IDA is usually decomposed based on IPAT formula or its derived formula, and only needs the conventional statistical data of each department therefore, it is simple to calculate and easy to apply. The logarithmic mean divisia index (LMDI) is a commonly used form of decomposition in IDA [25]. Jeong et al. [26], Tian et al. [27], Karakaya [28] used LMDI to analyse the driving factors of carbon emissions in South Korea, China and Turkey. Bianco et al. [29], Ozdemir et al. [30] used LMDI-Tapio model to decompose the carbon emissions and investigate the decoupling factors. However, IDA can only reflect the direct impact of various drivers on the indicators of concern, and cannot reflect the indirect impact caused by the interrelation of economic activities [31]. The SDA based on the input-output table considers the industrial characteristics, industrial ties and trade ties, which not only can calculate the direct driving effect of the final demand on the research indicators, but also analyse the indirect impact caused by the interrelation of various production sectors, and extend the depth of decomposition analysis. It is indeed a more complex and precise decomposition method [32–34]. Su et al. [35] used SDA to analyse the different results of four driving factors of China’s trade between 1997 and 2002 under the traditional input-output model and the improved input-output model. Wang et al. [8], Liu et al. [36], Hao & Liu [37] used SDA to explore the deep driving factors of China’s carbon emissions from different levels such as industrial structure, household consumption, international trade, etc., and found that the growth of China’s carbon emissions should be curbed by industrial upgrading, developing renewable energy and increasing foreign direct investment. Considering the effect of fiscal decentralisation on curbing carbon emissions, Xia et al [38] found that the local government should reduce the retained production tax to reduce the motivation of the government to tax pollution high energy consuming industries and increase carbon emissions. In addition, considering regional differences, Wang et al. [39] argued that the same driver has different effects on carbon emission intensity in different provinces of China.

In summary, the existing evidence on the determinants of carbon emission is mixed and far from conclusive. Therefore, conducting an in-depth analysis of determinants of carbon emissions in Gansu Province holds significant practical importance. The fresh insight gained from studying a least developed and energy intensive province will offer valuable perspective for achieving carbon mitigation in China as a whole. This research stands to contribute meaningful data to the broader understanding of effective strategies for carbon reduction, particularly in regions facing unique challenges such as Gansu province.

## 3. Research method

### 3.1 Methodology for measuring total carbon emissions

This paper examines carbon dioxide emissions from fossil energy combustion, the equation for measuring total carbon emissions is as follows:

$$CE=\left[\begin{array}{c} {ef}^{1}\\ \vdots \\ {ef}^{17} \end{array}\right]*[{en}^{1}\;\cdots \;{en}^{17}]$$ (1)

Eq (1) can be simplified as follows.


CE=EF*EN (2)

Included among these, the *CE* represents the Total Carbon Emissions, *EF* is the carbon dioxide emission factor, and the element in column j represents the carbon dioxide emission coefficient of the jth energy, matrix *EN* is the energy consumption matrix expressed in calorific value unit, and the element in line i represents the consumption of the ith energy. The total amount of carbon dioxide emissions can be obtained by multiplying the consumption of each energy by its carbon dioxide emission factor and summing it up.

### 3.2 Structural decomposition analysis methods

The Structural Decomposition Analysis (SDA) model is a suitable and popular tool to reveal the driving factors of carbon emission changes. Compared with IDA, SDA has higher data requirements, which is its main challenge. However, the main advantage of SDA is that it can comprehensively analyse all kinds of direct or indirect influences by virtue of the input-output model, especially the indirect influence of changes in demand in one sector on other sectors [31].

In Eq (2), the Energy Consumption Matrix *EN* can be further decomposed as follows:

$$EN=\left[\begin{array}{ccc} {es}^{1,1} & \cdots & {es}^{1,27}\\ \vdots & \ddots & \vdots \\ {es}^{17,1} & \cdots & {es}^{17,27} \end{array}\right]*\left[\begin{array}{c} {ei}^{1}\\ \vdots \\ {ei}^{27} \end{array}\right]\hat{\left[\begin{array}{c} {ei}^{1}\\ \vdots \\ {ei}^{27} \end{array}\right]}*\left[\begin{array}{c} x_{1}\\ \vdots \\ x_{27} \end{array}\right]$$ (3)

The Eq (3) simplifies to:

$$EN=ES*\hat{EI}*X$$ (4)

Included among these, *ES* refers to the energy structure, and the element in line i and column j represents the proportion of the i-th energy in the total energy use of the j-th sector. The higher the proportion of energy with higher carbon dioxide emission factor, such as raw coal, in the energy structure, the more unfavorable the carbon emissions would be. *EI* is energy intensity. The element in line i is the energy consumption of sector i divided by its sector output value. The higher the energy intensity is, the lower the energy efficiency is, which is not conducive to reducing carbon emissions. ^ represents diagonalising the matrix. *X is* the element in the i-th line that indicates the output value of the i-th sector.

In an input-output model *X* for.


$$X=\left[\begin{array}{ccc} a_{d}^{1,1} & \cdots & a_{d}^{1,27}\\ \vdots & \ddots & \vdots \\ a_{d}^{27,1} & \ldots & a_{d}^{27,27} \end{array}\right]*X+\left[\begin{array}{c} Y_{d}^{1}\\ \vdots \\ Y_{d}^{27} \end{array}\right]$$ (5)

The Eq (5) simplifies to:

$$X=A_{d}*X+Y_{d}$$ (6)

The Eq (6) simplifies to:

$$X={\left(I-A_{d}\right)}^{-1}*Y_{d}$$ (7)

*A*_*d*_ is a matrix of direct consumption coefficients for domestic products. (*I*−*A*_*d*_)^−1^ is the Leontief inverse matrix; the *Y*_*d*_ denotes the final demand matrix for the domestic product sector. *A*_d_*X representing the value of output of intermediate inputs in each sector. *y*_*d*_ represents the value of output used by final demand in each sector, which, when added together, equals the total value of output in each sector.

Matrices *Y*_*d*_ can be decomposed into.


$$Y_{d}=\left[\begin{array}{ccc} y_{str}^{1,1} & \cdots & y_{str}^{1,6}\\ \vdots & \ddots & \vdots \\ y_{str}^{27,1} & \cdots & y_{str}^{27,6} \end{array}\right]*\left[\begin{array}{c} y_{cat}^{1}\\ \vdots \\ y_{cat}^{6} \end{array}\right]*Y_{vol}$$ (8)

The Eq (8) simplifies to:

$$Y_{d}=Y_{str}*Y_{cat}*Y_{vol}$$ (9)

*Y*_*str*_ is sectoral structure for final needs. *Y*_*cat*_ is the type of structure of the final demand, the *Y*_*vol*_ for total demand.

Therefore,

$$CE=EF*ES*\hat{EI}*L_{d}*Y_{str}*Y_{cat}*Y_{vol}$$ (10)

Matrices *L*_*d*_ For Leontief inverse matrix, this paper assumes that the carbon dioxide emission factor EF remains constant.


$$CES=diag(EF*ES*\hat{EI}*L_{d})*Y$$ (11)

*CES* exhibits carbon emissions. *Y* is the final demand. *diag* Represents diagonalising the matrix. Eq (11) is possible to calculate the implied carbon emission caused by each final demand on the product demand of each sector, and analyse the carbon emission relationship between industry and demand in Gansu Province in a comprehensive manner.


$$CIS=diag(EF*ES*\hat{EI}*L_{d})*Y_{d}$$ (12)

$$CIC=EF*ES*\hat{EI}*L_{d}*Y_{str}$$ (13)

*CIS* The element in line i is the implied carbon emission intensity of sector i, *CIC* The element in column i is the implied carbon emission intensity of the i final demand. Through Eqs (12) and (13), the calculations enable better identification of priority sectors and priority final demand for emission reductions.

While considering the alternatives of addition principle and multiplication principle, since this paper studies the total carbon emissions of Gansu Province, which belongs to the total amount indicator, according to the research of Wang et al. [34], the total amount indicator is suitable for the addition principle, so this paper uses the addition principle to decompose the change of total carbon emissions into six driving factors, the similar calculation steps can be found in Su et al. [40]:

$$\Delta CE=EF*\Delta ES*{\hat{EI}}_{1}*L_{d,1}*Y_{str,1}*Y_{cat,1}*Y_{vol,1}$$

$$+EF*{ES}_{0}*\Delta \hat{EI}*L_{d,1}*Y_{str,1}*Y_{cat,1}*Y_{vol,1}$$

$$+EF*{ES}_{0}*{\hat{EI}}_{0}*\Delta L_{d}*Y_{str,1}*Y_{cat,1}*Y_{vol,1}$$

$$+EF*{ES}_{0}*{\hat{EI}}_{0}*L_{d,0}*\Delta Y_{str}*Y_{cat,1}*Y_{vol,1}$$

$$+EF*{ES}_{0}*{\hat{EI}}_{0}*L_{d,0}*Y_{str,0}*\Delta Y_{cat}*Y_{vol,1}$$

$$+EF*{ES}_{0}*{\hat{EI}}_{0}*L_{d,0}*Y_{str,0}*Y_{cat,0}*\Delta Y_{vol}$$ (14)

The subscript 0 indicates the starting year and the subscript 1 indicates the ending year. The first term to the right of the equal sign represents the energy structure effect, the second term represents the energy intensity effect, the third term represents the input-output structure effect, the fourth term represents the demand sector structure effect, the fifth term represents the demand type structure effect, and the sixth term represents the total demand effect. The above approach refers to the research of Yan et al. [41].

However, there are many decomposition forms of the above formula. For LMDI and D&L methods, Cao et al. [42] used D&L, while Zhang et al. [43] used LMDI. Although LMDI is simpler, the research and analysis of Boer et al. [44] shows that LMDI will be very cumbersome when there are zero or negative values in the input-output table, D&L has better potential to deal with zero and negative values in the input-output table although it has a large amount of calculation, therefore, this paper chooses the D&L method. That is:

$$\Delta CE=E(\Delta ES)+E(\Delta \hat{EI})+E(\Delta L_{d})+E({\Delta Y}_{str})+E(\Delta Y_{cat})+E(\Delta Y_{vol})$$ (15)

$$E(\Delta x_{i})=\sum_{s}f(s)*EF*\prod_{j=1,j\neq i}^{n}x_{j,time}(\Delta x_{i})$$ (16)

Where time = 0 or 1, Σ_S_ means right {x_j,time_|J = 1,.,n and j≠i} Sum all combinations of time in, s is the number of combinations of time = 1, and *f*(*s*) = *s*!(*n*−*s*−1)!/*n*!. By Eqs (15) and (16), the amount of change in carbon emissions can be fully and efficiently disaggregated into the six drivers.

### 3.3 Structural decomposition analysis of carbon emissions in various industrial sectors

Energy structure effect. The 17*27 matrix ΔES_(j)_ is a matrix that only retains ΔES the elements in column j, and the other elements are all 0 ($\Delta ES=\sum_{j=1}^{27}{\Delta ES}_{\left(j\right)}$). Then the energy structure effect in sector j can be expressed as E(ΔES_(j)_).


$$E({\Delta ES}_{\left(j\right)})=\sum_{s}f(s)*EF*{\Delta ES}_{\left(j\right)}*{\hat{EI}}_{time}*L_{d,time}*Y_{str,time}*Y_{cat,time}*Y_{vol,time}$$ (17)

Energy intensity effect. The 27*1 matrix ΔEI_(i)_ is a matrix that only retains ΔEI the elements in row i, and the other elements are all 0 ($\Delta EI=\sum_{i=1}^{27}{\Delta EI}_{\left(i\right)}$). The energy intensity effect of sector i can be expressed as $E(\Delta {\hat{EI}}_{\left(i\right)})$.


$$E(\Delta {\hat{EI}}_{\left(i\right)})=\sum_{s}f(s)*EF*{ES}_{time}*\Delta {\hat{EI}}_{\left(i\right)}*L_{d,time}*Y_{str,time}*Y_{cat,time}*Y_{vol,time}$$ (18)

Input-output structure effect. The 27 *27 matrix ΔA_d(j)_ is a matrix that only retains ΔA_d_ the elements in column j, and the other elements are all 0 ($\Delta A_{d}=\sum_{j=1}^{27}{\Delta A}_{d(j)}$). Because ΔL_d_ = L_d,1_ΔA_d_L_d,0_, the input-output structure effect of sector j can be expressed as E(L_d,1_ΔA_d_L_d,0_).


$$E(L_{d,1}\Delta A_{d}L_{d,0})=\sum_{s}f(s)*EF*{ES}_{time}*{\hat{EI}}_{(i),time}*L_{d,1}\Delta A_{d}L_{d,0}*Y_{str,time}*Y_{cat,time}*Y_{vol,time}$$ (19)

Demand sectoral structure effect. The 27*6 matrix ΔY_str(i,j)_ is a matrix that only retains ΔY_str_ the element in row i and in column j, and the other elements are all 0 ($\Delta Y_{str}=\sum_{j=1}^{6}\sum_{i=1}^{27}{\Delta Y}_{str(i,j)}$). The demand sectoral structure effect of sector j can be expressed as $\sum_{j=1}^{6}E({\Delta Y}_{str(i,j)})$.


$$E({\Delta Y}_{str(I,j)})=\sum_{s}f(s)*EF*{ES}_{time}*{\hat{EI}}_{(I),time}*L_{d,time}*{\Delta Y}_{str(I,j)}*Y_{cat,time}*Y_{vol,time}$$ (20)

Demand category structure effect. The 6*1 matrix ΔY_cat(i)_ is a matrix that only retains ΔY_cat_ the element in row i, and the other elements are all 0 ($\Delta Y_{cat}=\sum_{i=1}^{6}\Delta Y_{cat(i)}$). Let $\Delta D_{Y_{cat(i)}}=Y_{str}*\Delta Y_{cat(i)}*Y_{vol}$. The 27*1 matrix $\Delta D_{Y_{cat(i)}}$ is the change in the final demand i of each sector caused by the change in proportion of final demand i in the total final demand. The demand category structure effect of sector j is the element in column j of $\sum_{i=1}^{6}E(\Delta {\hat{D}}_{Y_{cat(i)}})$.


$$E(\Delta {\hat{D}}_{Y_{cat(i)}})=\sum_{s}f(s)*EF*{ES}_{time}*{\hat{EI}}_{(i),time}*L_{d,time}*\Delta {\hat{D}}_{Y_{cat(i)}}$$ (21)

6. Demand volume effect. Let $\Delta D_{Y_{vol(i)}}=Y_{str}*Y_{cat(i)}*{\Delta y}_{vol}$. The 27*1 matrix $\Delta D_{Y_{vol(i)}}$ is the change in demand of each sector of final demand i caused by the change in total final demand volume. The demand volume effect of sector j is the element in column j of $\sum_{i=1}^{6}E(\Delta {\hat{D}}_{Y_{vol(i)}})$.


$$E\left(\Delta {\hat{D}}_{Y_{vol(i)}}\right)=\sum_{s}f(s)*EF*{ES}_{time}*{\hat{EI}}_{(i),time}*L_{d,time}*\Delta {\hat{D}}_{Y_{vol(i)}}$$ (22)

Eqs (17)–(22) can decompose the driving factors of carbon emission changes in sectors, which is helpful for detailed analysis of the internal contradictions in sectors’ carbon emissions.

## 4. Data sources and processing

### 4.1 Data sources

The energy inventory data of Gansu Province was collated by the China emission accounts and datasets (CEADs) team, which covers 17 kinds of energy consumption of 47 departments in Gansu Province. In terms of division of sectors, CEADs divided the final energy consumption of the energy balance sheet in the China Energy Statistical Yearbook into eight sectors: "agriculture, forestry, animal husbandry, fishery and water conservancy", "industry", "construction", "transportation, warehousing, post and telecommunications services", "wholesale, retail trade and catering", "other service sectors", "urban residents’ energy use" and "rural residents’ energy use", According to the Industrial Sector Energy Consumption Table (ISECT), the "industrial" sector was expanded to 40 sub sectors, resulting in 47 sectors. The selection of energy types is based on 26 kinds of fossil fuels in China’s energy data, integrating energy types with small consumption and similar quality, and finally 17 energy types are obtained. The energy consumption of each department is collected and calculated from the corresponding statistical yearbook of Gansu Province [45]. In this paper, in order to study the recent carbon emissions from energy consumption in Gansu Province, energy inventory data for 2007, 2012 and 2017 were selected.

Net calorific value of energy and carbon dioxide emission factor were determined by data of Shan et al. [46] from Intergovernmental Panel on Climate Change (IPCC) (see Table 1).

**Table 1:** Net caloric value and carbon emission factor of energy.

Code	Energy type	Net caloric value (PJ/10^4^t, 10^8^m^3^)	Carbon emission factor (t CO_2_/PJ)
**1**	**Raw Coal**	0.28	92708
**2**	**Cleaned Coal**	0.27	96301.33
**3**	**Other Washed Coal**	0.27	96301.33
**4**	**Briquettes**	0.26	92708
**5**	**Coke**	0.28	104925.3
**6**	**Coke Oven Gas**	1.88	43923
**7**	**Other Gas**	1.88	43923
**8**	**Other Coking Products**	0.43	93654
**9**	**Crude Oil**	0.42	72600
**10**	**Gasoline**	0.44	68607
**11**	**Kerosene**	0.44	70785
**12**	**Diesel Oil**	0.43	73326
**13**	**Fuel Oil**	0.40	76593
**14**	**LPG**	0.47	62436
**15**	**Refinery Gas**	0.50	56991
**16**	**Other Petroleum Products**	0.40	72600
**17**	**Natural Gas**	3.44	55539

### 4.2 Data processing

This paper follows Liang et al. [47] article and International Standard Industrial Classification of All Economic Activities (ISIC) to combine the 47 sectors in the input-output table into 27 sectors. First, the overlap of the input-output table sectors in different years is determined, then the cross-over overlap of the input-output table and energy list sectors is summarised until we arrive at a unified classification containing 27 sectors (see Table 2).

**Table 2:** Sector aggregation.

Code	Sector
**1**	Agriculture, forestry, animal husbandry and fishery
**2**	Coal mining and washing industry
**3**	Oil and gas extraction industry
**4**	Metal mining and dressing industry
**5**	Mining and dressing of non-metallic ores and other minerals
**6**	Food manufacturing and tobacco processing industry, wine, beverage and refined tea manufacturing
**7**	Textile industry
**8**	Textiles, garments, shoes, hats, leather, down and its products
**9**	Wood working and furniture manufacturing
**10**	Paper, printing and cultural, educational, sporting goods manufacturing and other manufacturing and scrap, metal products, machinery and equipment repair services
**11**	Petroleum processing, coking and nuclear fuel processing industries
**12**	Chemical industry and pharmaceutical manufacturing, chemical fiber manufacturing, rubber and plastic products industry
**13**	Non-metallic mineral products industry
**14**	Metal smelting and rolling processing industry
**15**	Metal products industry
**16**	General and special equipment manufacturing
**17**	Transportation equipment manufacturing
**18**	Electrical machinery and equipment manufacturing
**19**	Communication equipment, computer and other electronic equipment manufacturing
**20**	Instrumentation and cultural office machinery manufacturing
**21**	Electricity and heat production and supply industry
**22**	Gas production and supply industry
**23**	Water production and supply industry
**24**	Construction industry
**25**	Transportation, warehousing and postal services, information transmission, computer services and software industries
**26**	Wholesale and retail trade and accommodation and catering trade
**27**	Other

For the treatment of the energy inputs of thermal power and heat in the energy list, according to ISIC, the sector of electricity, steam and hot water production and supply includes the production of steam and hot water as well as electricity through boilers and other installations utilising energy sources such as coal, oil, gas, and so on. Therefore, in this paper, we treat the energy inputs of thermal power and heat in the energy list as the direct energy consumption of the sector of electricity, steam, and hot water production and supply, which facilitates a more detailed and complete analysis of the sector’s carbon emission.

In terms of the energy input of thermal power and heat power in the energy list, this study regards the energy input of thermal power and heat power as the direct energy consumption of power, steam and hot water production and supply sectors. This approach facilitates a more detailed and comprehensive analysis of the carbon emissions within the sector. Regarding the processing of inflows, which includes international imports and interprovincial imports, the data collected for this study utilises a competitive import input-output table. However, using data directly could lead to an overestimation of implied carbon emissions from exports [43]. Therefore, a non-competitive import input-output table is constructed, based on Weber et al. [46]. In the methodology used, it is assumed that the proportion of inflows in intermediate and final demand (excluding outflows) in each sector is the same as the average proportion of inflows in the corresponding sector. The inflows are subtracted proportionately from the demand in each sector to avoid errors.

## 5. Discussion and analysis

### 5.1 Decomposition analysis of total carbon emissions

**Fig 1:**
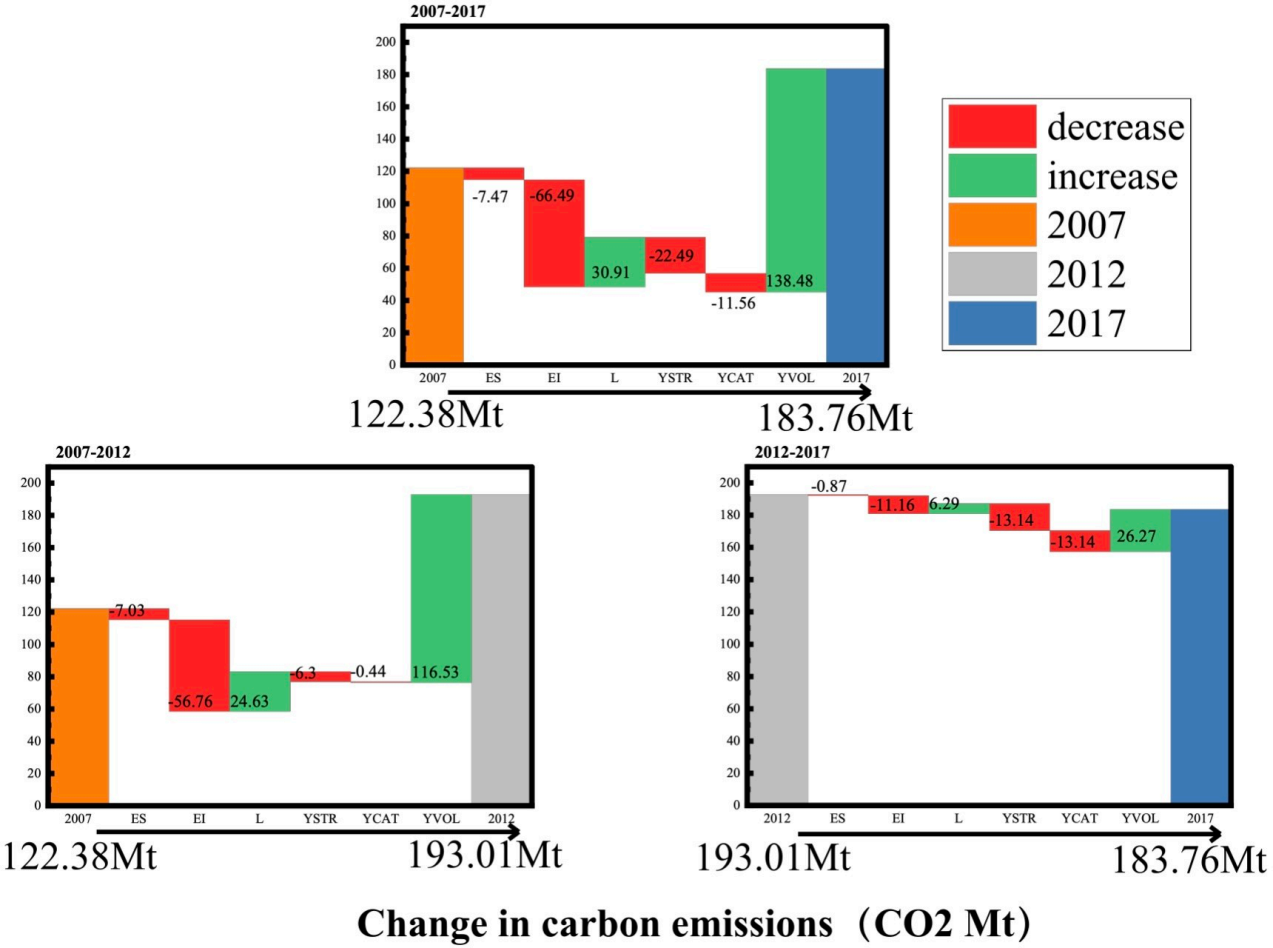
Total carbon emissions and their driving factors in Gansu Province.

Based on the input-output model, the total carbon emissions of Gansu Province in 2007, 2012 and 2017 and the structural decomposition analysis results of the changes of carbon emissions from 2012 to 2017 are presented in Fig 1. Calculated from Eqs (1)–(10), carbon emissions in Gansu Province showed an overall upward trend from 2007 to 2017, increasing from 122.38Mt in 2007 to 183.76Mt in 2017, an increase of 50.2%. This trend can be attributed to several factors. Firstly, since entering the 21st century, due to its weak economic foundation, Gansu Province has long regarded the expansion of industrial scale as its main economic growth point, that is, a rough economic development strategy. This approach has accelerated the development of high energy-consuming and high-emission industries, thus carbon emissions have continued to increase rapidly. Secondly, the rapid advancement of the tertiary industry in Gansu Province, paralleling a shift in consumption preferences from a product-centric approach to an emphasis on service. This shift has contributed to a modest reduction in the total carbon emissions from 2012 to 2017, declining from 193.01 Mt in 2012 to 183.76 Mt in 2017. It is crucial to highlight that this reduction is intricately tied to policy constraints and successful implementation of high-quality economic transformation and energy conservation measures, particularly since the 18th CPC National Congress and the energy conservation and emission reduction goals [47].

Eqs (14)–(16) can be used to obtain the contribution of the six drivers to the change of total carbon emissions in Gansu Province from 2007 to 2012, from 2012 to 2017, and from 2007 to 2017. Among the six driving factors influencing carbon emissions, the energy structure effect (ES) has a relatively small impact across all time periods. Overall, it has a negative effect, leading to a reduction of emissions by 7.47Mt, indicating a slight improvement in the overall energy structure of Gansu Province. Energy intensity effect (EI) exhibits a consistently negative impact on carbon emission across all time periods, especially during 2007 to 2012 period. During the 2007 to 2012 period, the reduction in emission amounted to 56.76Mt, EI standing out as the primary contributor to overall emission reduction. These changes imply that in 2007–2017 the efficiency of energy use in Gansu Province was improved and the structure of energy consumption was improved. On the contrary, the input-output structure (L) manifests a positive effect on carbon emission across all time periods. The positive effect of input-output structure underscores the extensive nature of Gansu Province’s economic development. The demand sector structure effect (YSTR) consistently contributed to emission reduction across all periods, with a notable emission reduction of 16.64Mt observed from 2012 to 2017. The overall improvement in the demand sector structure positions it as the second largest contributing factor to emission reduction. The demand type structure effect (YCAT) has a negative effect on emission reduction across all time periods. The total demand effect (YVOL) has a positive effect across all time periods, particularly evident from 2007 to 2012, where a substantial increase of 116.53 Mt in emissions occurred. The YVOL stands out as the most significant factor contributing to overall emissions, which indicates that economic development in Gansu Province was still the primary factor driving the growth of carbon emissions.

### 5.2. Carbon emission analysis of final demand and sectors

**Fig 2:**
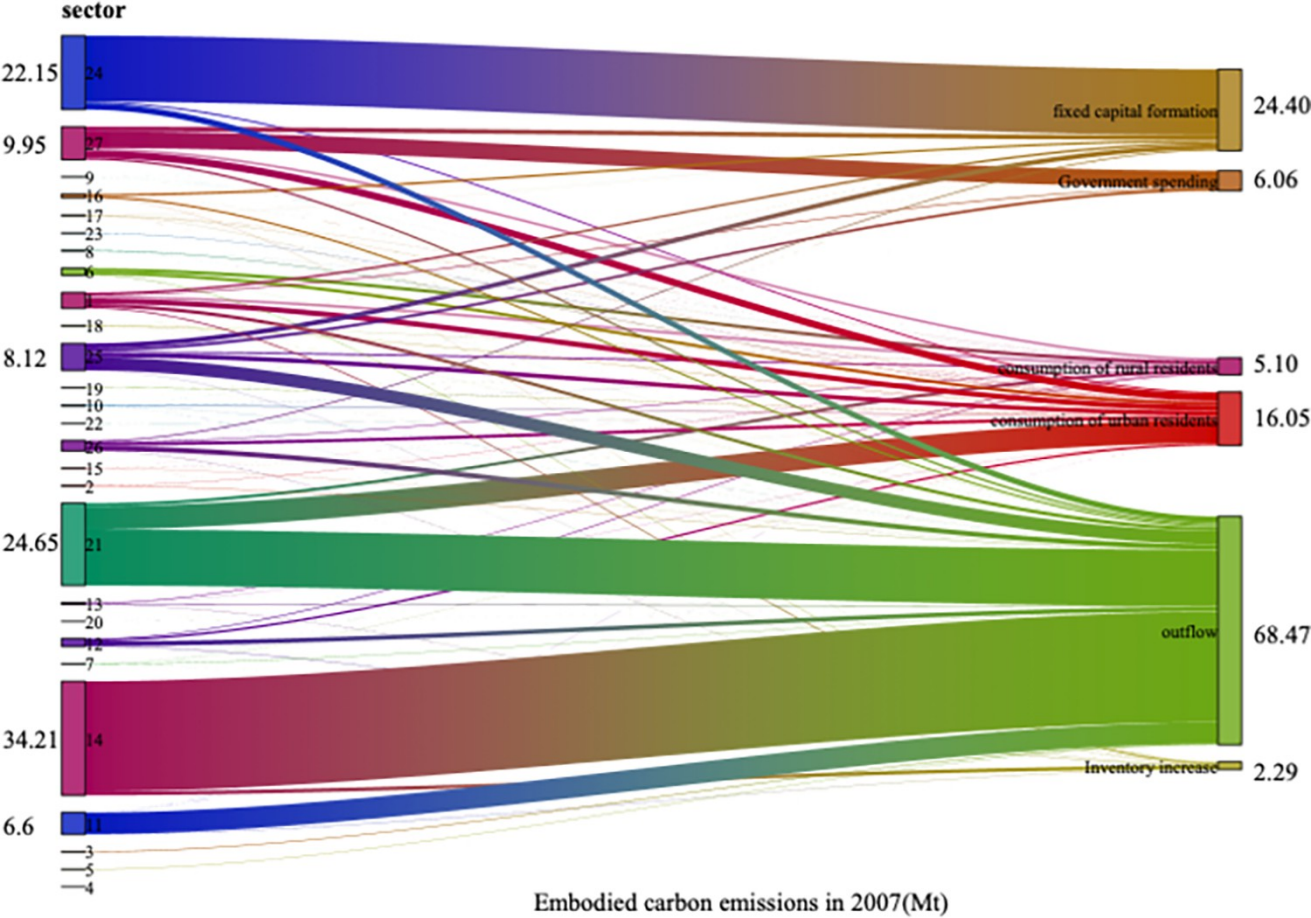
Carbon emission caused by different final demands from different sectors in 2007.

**Fig 3:**
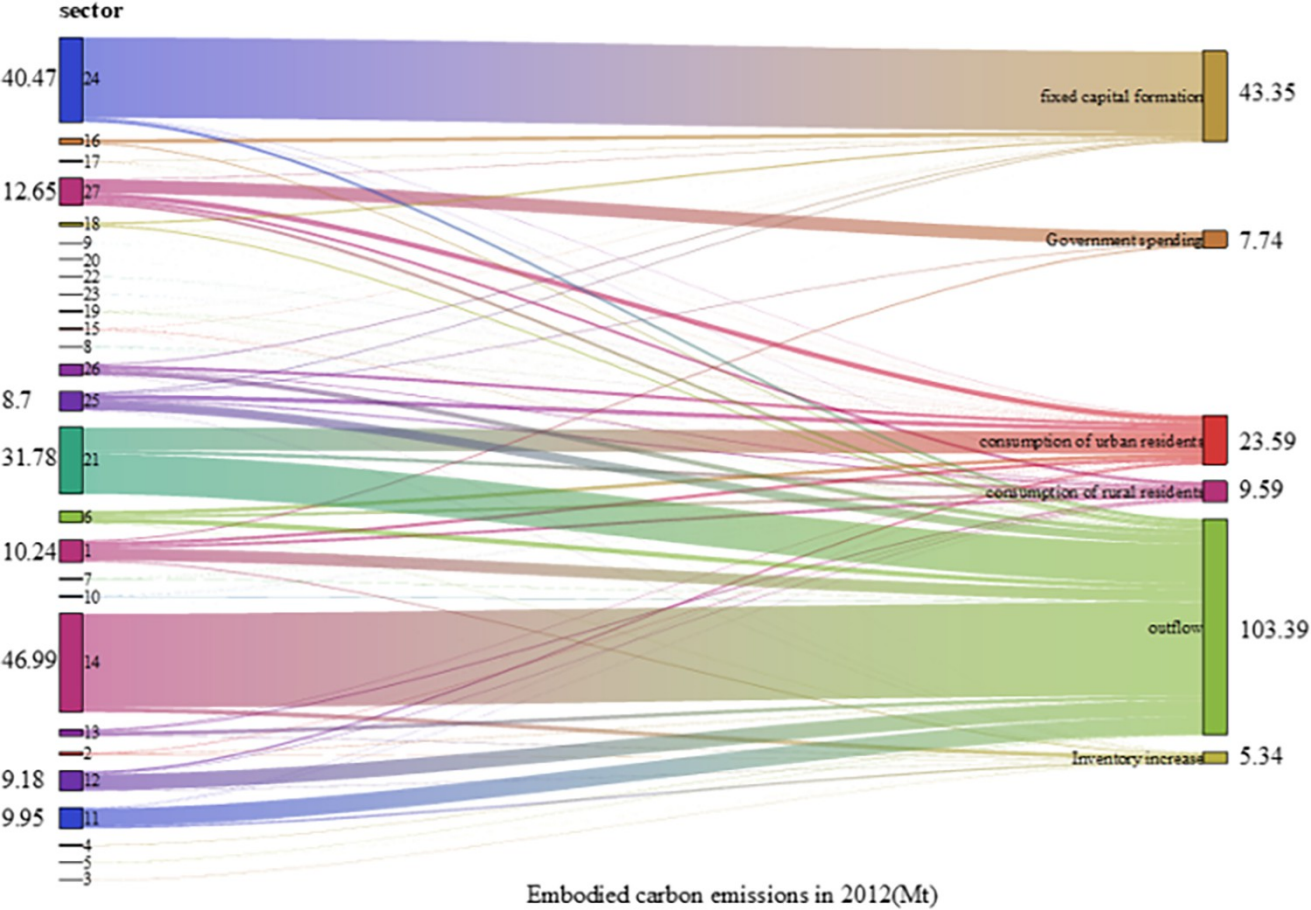
Carbon emission caused by different final demands from different sectors in 2012.

**Fig 4:**
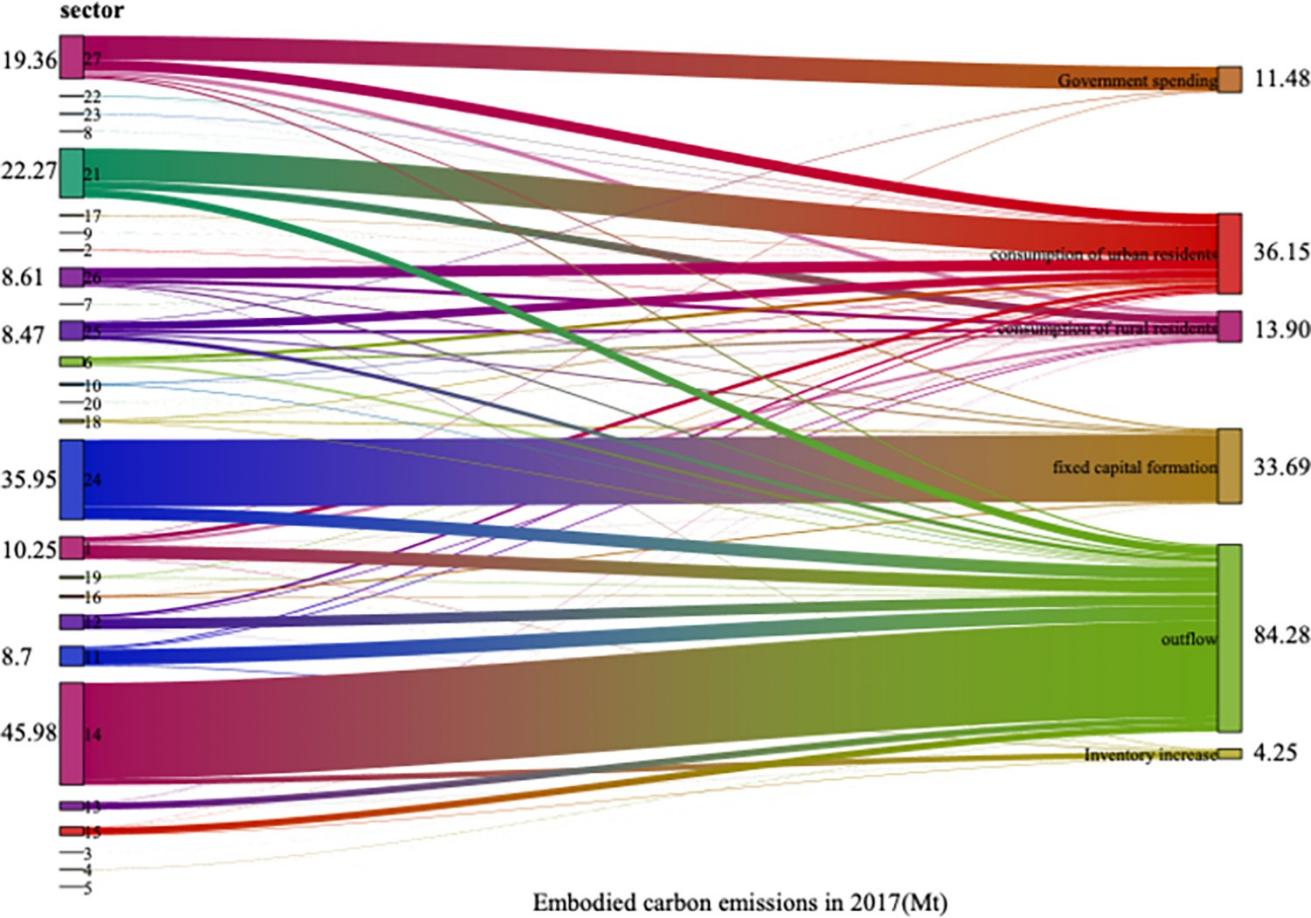
Carbon emission caused by different final demands from different sectors in 2017.

**Fig 5:**
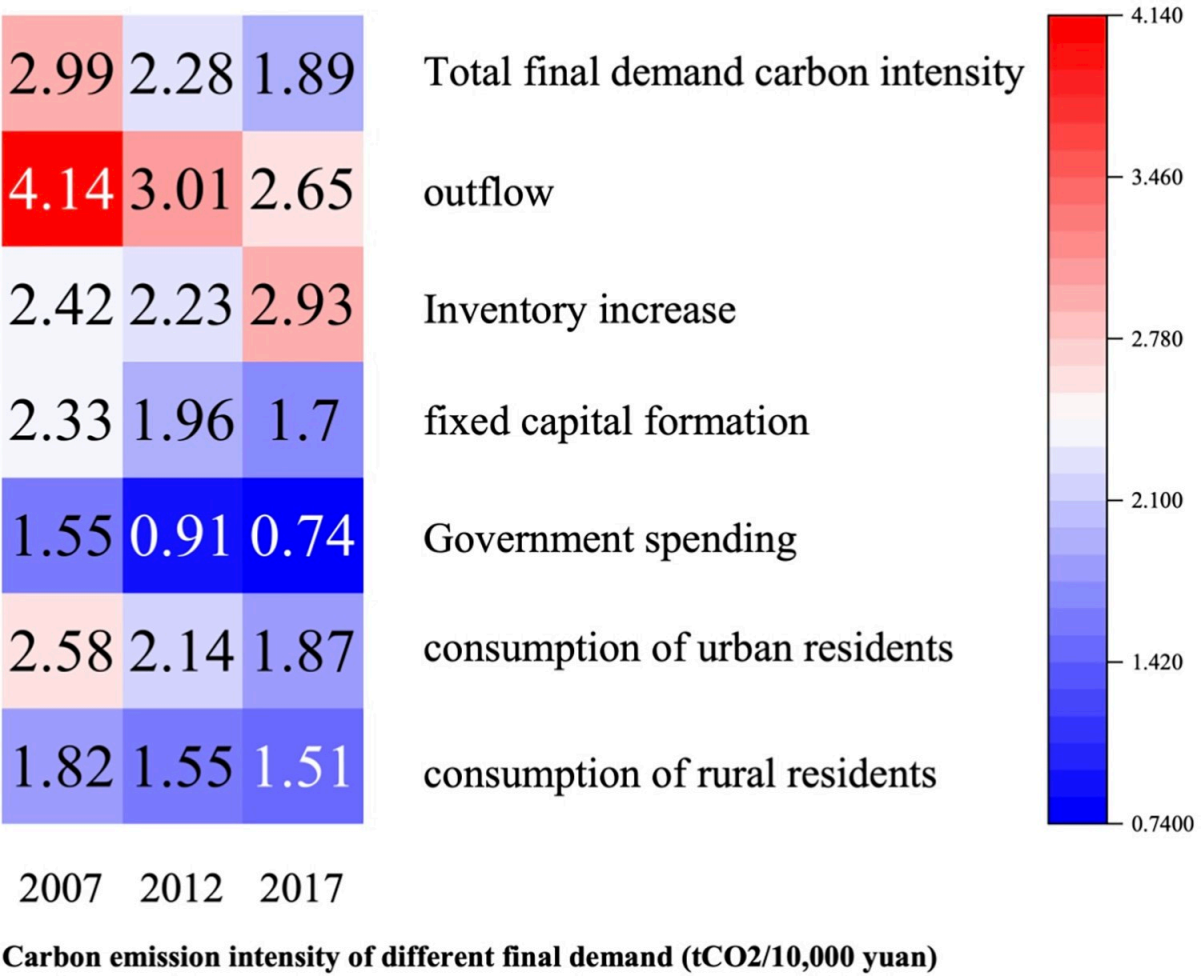
Carbon intensity of final demand.

The relationship between final demand and sectors carbon emissions, as well as the carbon intensity of final demand and sectors in 2007, 2012 and 2017, was computed using Eqs (13)–(15) As illustrated by Figs 2–5, the total final demand carbon intensity decreased from 2.99 tCO2/10,000 yuan in 2007 to 1.89 tCO2/10,000 yuan in 2017, with all category of final demand carbon intensities generally showing a year-on-year downward trend. Analysing each final demand category, it can be seen that outflow’s contribution to carbon emission is the highest, accounting for 56.0%, 53.6% and 45.9% of the emission in 2007, 2012 and 2017 respectively. Despite a decline in its proportion, outflow remains the largest contributor annually, highlighting Gansu Province’s role as a major energy supplier to other regions in China. Similarly, the carbon emission from urban residents’ consumption have significantly contributed to the overall carbon footprint, accounting for 13.1%, 12.2% and 19.7% of carbon emission in 2007, 2015 and 2017 respectively. This illustrates a consistent upward trajectory. The carbon emission caused by final demand increased by 20.1Mt from 2007 to 2017. The formation of fixed capital also has a great impact on the total carbon emissions, accounting for 18.3% in 2017. From 2007 to 2012, the carbon emissions of fixed capital formation increased by 18.95Mt, and from 2012 to 2017, it decreased by 9.66Mt. Overall, from 2007 to 2017, it increased by 9.29Mt, increasing the total carbon emissions. The consumption of rural residents contributed little to carbon emissions, accounting for 7.6% in 2017, showing an overall increasing trend. Government expenditure made a small contribution to carbon emissions, accounting for 6.2% in 2017. From 2007 to 2017, the carbon emissions caused by government expenditure increased by 5.41Mt. The total amount and change of carbon emissions due to inventory increase are small, but the intensity of carbon emissions is high.

**Fig 6:**
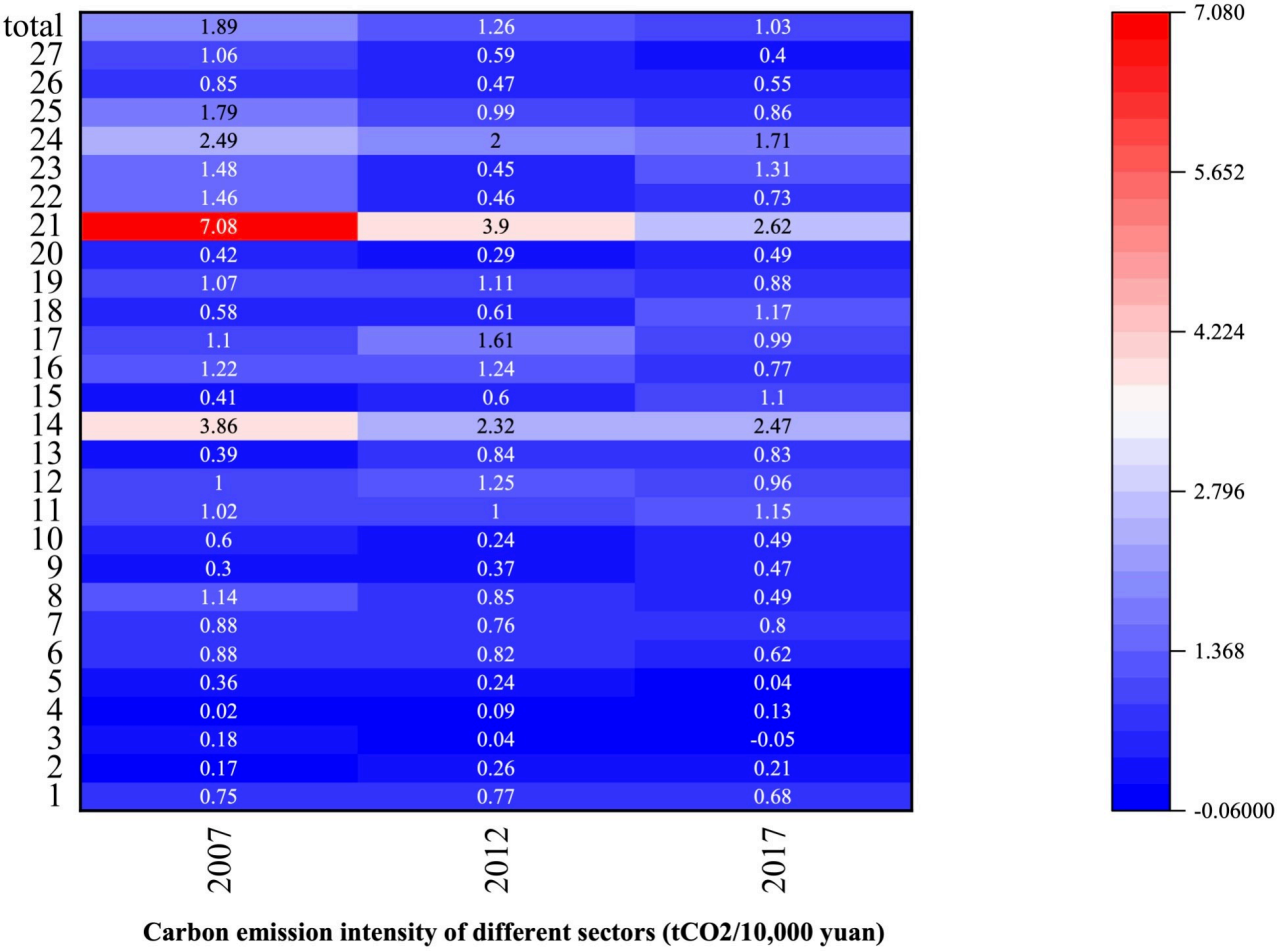
Carbon intensity of sectors.

Sectoral implied carbon emissions refer to the carbon emissions generated by specific department in the entire production chain, involved in manufacturing products and providing services [48]. For example, the production of automobiles necessitates the consumption of materials such as iron and steel, and the carbon emissions originating from the production of that iron and steel are integrated into the implied carbon emissions associated with automobile. According to Figs 2–4, Metal Smelting and Rolling Processing Industry (sector 14), had the largest implied carbon emissions, followed by Construction Industry (sector 24). This underscored the imperative for Gansu Province to orient its development strategies towards low-carbon practices, particularly with the energy sector and the constructions industry. As Fig 6 showed the overarching trend of decreasing sectoral carbon emission intensity over the years, plummeting from 1.89 tCO_2_/10000 yuan in 2007 to 1.03 tCO_2_/10000 yuan in 2017. Electricity and Heat Production and Supply Industry (sector 21) exhibited the highest carbon emission intensity, despite a discernible trend, it was 2.62 tCO_2_/10000 yuan in 2017. Metal Smelting and Rolling Processing Industry (sector 14) took the second place. After the decline from 2007 to 2012, carbon emission intensity recovered from 2012 to 2017, reaching 2.47 tCO_2_/10000 yuan in 2017. Construction Industry (sector 24) also demonstrates relatively high carbon emission intensity. Although there is a yearly decrease, it remained elevated in 2017 at 1.71 tCO_2_/10000 yuan. The implied carbon emissions of Oil and Gas Extraction Industry (sector 3) were negative in 2017, as the inventory increase in the 2017 input-output table was negative, and the final use was also negative.

### 5.3 Decomposition analysis of carbon emission drivers by sector

**Fig 7:**
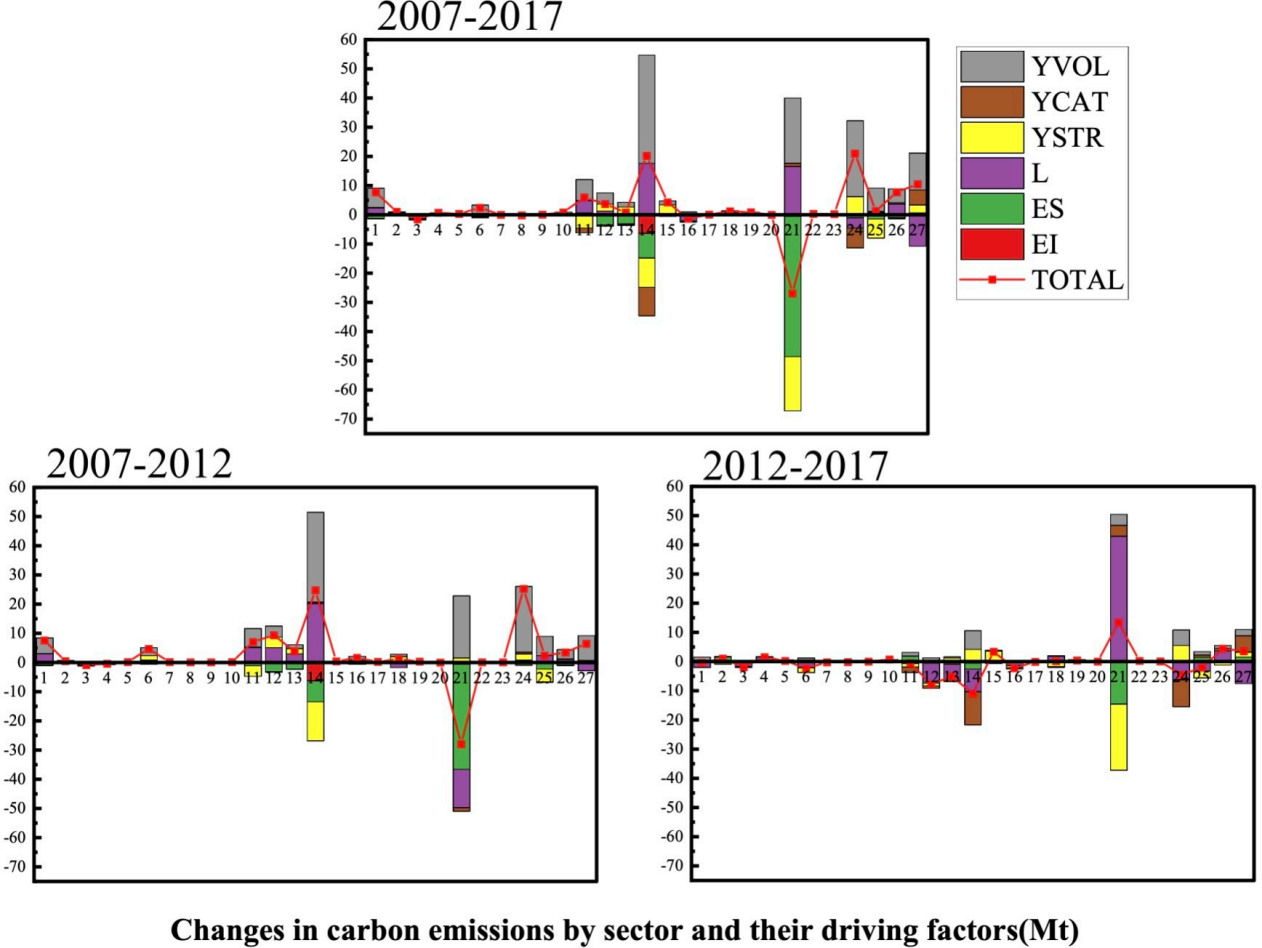
Structure decomposition analysis of each sector’s total emission in Gansu. Note: YVOL means the total demand effect, YCAT means the demand type structure effect, YSTR means the demand sector structure effect, L means the input-output structure, ES means energy structure effect, EI means energy intensity effect.

Using Eqs (17)–(22) we calculated the amount of changes in carbon emissions and their driving factors in each sector in Gansu Province from 2007 to 2012, 2012 to 2017 and 2007 to 2017. The results are depicted in Fig 7 Electricity and Heat Production and Supply Industry (sector 21) experienced the most significant alterations in carbon emissions, witnessing a reduction of 26.95 Mt of carbon emissions from 2007 to 2017. Moreover, the energy intensity effect and the demand sector structure effect are the main emission reduction factors, while the total demand effect and the input-output structure effect are the main emission increase factors. From 2012 to 2017, the total carbon emissions of sector 21 increased, with the input-output structure effect playing a predominant role in this carbon emission surge, contributing an increase of 42.90Mt. Metal Smelting and Rolling Processing Industry (sector 14) took the second place. From 2007 to 2017, carbon emissions in this sector increased by 20.25 Mt. The major contributors to this increase were identified as the total demand effect and input-output structure effect, while other driving factors played a role in reducing emissions. A more in-depth analysis of sector 14 revealed that, despite a decrease of 11.00Mt in the second half of the period, the cumulative effects of emission increase from 2007 to 2012 could not be completely offset.

Construction Industry (sector 24) had undergone significant changes in total carbon emissions. Overall, this sector witnessed an increase of 20.97 Mt, with total demand effect playing the most dominant role in this carbon emission increase, followed by the demand sector structure effect. Although the total demand effects continued to contribute to emission increases, however, the magnitude of the increase notably diminished. On the contrary, the effects demand sector structure on carbon emission intensified over time. The other sectors (sector 27) have shown an increase in carbon emission across all time periods. Overall, the total demand effect played a major role in increasing emissions, while the input-output structure effect was effective in reducing emissions. Wholesale and Retail Trade and Accommodation and Catering Trade (sector 26) consistently played an increasingly pivotal role in emissions across all time periods. In contrast, Electricity and Heat Production and Supply Industry (sector 21) improved their carbon emissions, while the total carbon emissions of other sectors continued to increase or stayed the same. From a sectoral perspective, each driving factor assumed a distinct role in different sectors. In general, the energy intensity effect contributed to energy conservation and emission reduction, while the total demand effect played a role in increasing emissions, which is consistent with the conclusion obtained from the decomposition of total carbon emissions in the previous section.

## 6. Conclusions and recommendations

Based on the input-output model, this paper used SDA to calculate the carbon emissions of each final demand from each sector and revealed the driving factors of carbon emission changes in Gansu Province from 2007 to 2017. The ensuing conclusions are as follows:

The primary contributors to emission increase are the total demand effect and the input-output structure effect, while the energy intensity effect and the demand sector structure effect are the most important factors for emission reduction. The economic development of Gansu Province in 2007–2017 significantly contributed to the increase of carbon emissions due to poor economic development practices. Moreover, the trend of rough economic development persists and continues to deteriorate, posing challenges to the effective implementation of emission reduction measures. On the positive side, optimising the efficiency of energy use is recognised as a favorable approach to reducing carbon emissions.

Outflow is the ultimate demand responsible for the total carbon emissions and carbon intensity. Similarly, consumption and fixed capital formation by urban residents make significant contribution to total carbon emissions. Rural residents’ consumption contributes less to carbon emissions, but there is ample room for optimising carbon emission intensity. The increase of inventory contributes less to the total carbon emission, but the carbon emission intensity is high. It can be seen that Gansu Province, while actively participating in international trade, should also pay attention to the reduction of stockpiles.

Metal Smelting and Rolling Industry, Electricity and Heat Production and Supply Industry and Construction Industry are identified as key sectors with high carbon emissions and carbon intensity, requiring focused attention for emission reduction. Metal Smelting and Rolling Industry is the most important sector in terms of emission increase, and the total demand effect and the input-output structure effect are the main factors responsible for emission increase. Carbon emissions from Electricity and Heat Production and Supply Industry have improved, but the total carbon emissions and carbon emissions intensity are high, and the total demand effect and input-output structure effect are the main factors for emission increase. The development mode of Metal Smelting and Rolling Industry as well as Electricity and Heat Production and Supply Industry need to be optimised. Construction Industry is the main emission-increasing sector, with the total demand effect and the structural effect of the demand sector being the main emission-increasing factors.

Based on the above main conclusions, in order to achieve the carbon neutrality goal, this paper puts forward the following suggestions: 1) For the input-output structure effect, the government should optimise the industrial structure, change the mode of economic development and actively develop green and ecological industries. 2) For the energy intensity effect, the government is supposed to deal with the relationship between energy development and ecological protection, accelerate the greening and intelligent development of energy development and promote the clean and efficient use of coal. 3) For the key sectors of emission reduction, the government should promote the key industry enterprise pollution control, strengthen the transformation and upgrading of traditional industries. 4) Gansu Province should aim at reducing key end-use demand, active integration into international trade, and realising the imperativeness of high-quality trade development.

## Supporting information

S1 File (ZIP)

S2 File (ZIP)

S3 File (ZIP)

S4 File (ZIP)

S5 File (ZIP)

S6 File (ZIP)

S7 File (ZIP)

S8 File (ZIP)
